# The National Milk Supply

**Published:** 1904-05-14

**Authors:** 


### The Hospital.

A Journal of

The Medical Sciences and Hospital Administration.

Vol. XXXVI.—No. 920. MAY 14, 1904.

### THE NATIONAL MILK SUPPLY.

Sir James Crichton-Browne is always original; and, besides being original, is gifted with the happy knack of leading an audience away from details to the consideration of wider issues. His Presidential Address to the Association of Sanitary Inspectors, delivered on Saturday last, furnished an admirable illustration of the exercise of the powers to which we have referred; for, although speaking to an audience the members of which are primarily concerned with milk only in relation to the dilutions or adulterations practised by retail dealers, he succeeded in guiding their thoughts to its physiological position as a food, and to the national importance of augmenting the quantity and of protecting the quality of the supply. It is, unfortunately, only too notorious that the action of the Board of Agriculture, in fixing a medium standard as the normal proportion of butter fat in milk, has led to a larger and more systematic dilution than has ever before been practised; the present aim of dealers being to reduce all the better class of milks to the legalised quality, and this aim being accomplished by careful measuring and testing in large establishments, rather than by the casual or haphazard methods of the small shopkeeper. An evil which the law has produced is one for which the same agency may be expected in time to provide a remedy; but, in order that this may be done, the first essential is to educate the public to the real value of milk as food, and to the degree in which the dishonesty of a particular class of traders may affect the general welfare. Sir James, for this purpose, took up his parable at the beginning, and dwelt with great force upon the disastrous consequences to infants of their being deprived of their natural food from the maternal breast. He described the extent to which mothers now shirk their duties in this respect as one of the most lamentable and disquieting signs of the times; and, while admitting that many women nowadays are constitutionally unequal to lactation, declared it to be one of the indications of the degeneracy which prevails that the capacity for nursing is steadily decreasing among women of the cultured classes. Besides the examples of this decrease there are, he declared, regiments of women who merely dislike the restrictions and disabilities which nursing imposes, and who seek to escape from them by any frivolous excuses. Of the consequences to the vitality of children he drew a pitiable picture, according to which, of the 150,000 infants who die annually in this country in the first year of life, three-fourths have been fed artificially and only one-fourth have been nursed by their mothers. In France, he tells us, the mortality of breast-fed children is 8 per cent., while that of hand-fed children is 61 per cent.; and, however much the truth may be disguised under the names of assigned causes of death, such as diarrhœa, dysentery, gastro-enteritis, and so forth, the true cause is always malnutrition from improper food. There can be no question, he says, that innocents are to day being slaughtered on a scale that Herod would have considered atrocious. Various forms of advertised food for infants receive naturally the condemnation they so justly merit. Thousands of infants, Sir James declares, have died of them; thousands are being maimed for life by their deficiencies.

Sir James enters into a curious calculation of the pecuniary cost of abstinence from maternal nursing by asserting that the normal milk-supply of English mothers for the year 1901, calculated upon the birth-rate, would have been 63 million gallons, or about one-eighth of our whole supply of cow's milk, and worth, at a shilling a gallon, £3,150,000. How much of this was utilised for the benefit of their offspring or how much was wasted or suppressed it is, of course, impossible to say; but, none the less, the figures are striking and suggestive. They add force to the declaration that it should be regarded as disgraceful for the woman of means and of robust constitution to "forget her sucking child," or to imperil its well-being by her dissipations. Sir James appeals to the members of the medical profession to exert their influence in the matter, and to refuse their countenance to indolent, evasive and trivial excuses for non-nursing; but he should rather, we think, address his appeal to women occupying the position of leaders in fashionable society. Many of these can neither be wholly dead to the maternal instinct nor wholly absorbed in the frivolities to which their lives appear to be devoted. Many would possibly be glad of an occasional or even periodical escape from the treadmill of their customary existence; and, if such could be induced to lead, Suburbia would hasten to follow.

In the meanwhile, and pending the arrival of a time when the mothers of England will be more sensible of their responsibilities and better able than at present to discharge them, Sir James points out that a very important addition to the national milk supply, and an addition which might be rendered speedily available for the children of cottagers and of the poor, would be obtained by increased attention to the rearing and utilisation of goats, animals which already yield a large proportion of the milk consumed in many other countries, and which will thrive either on the scanty herbage of the bare cliffs around our coasts, on our scrubby uplands, on railway embankments, and on the patches of coarse grass by the roadsides, on the refuse of gardens, or almost equally well when stall fed. The goat yields, he tells us, sometimes as much as four quarts of milk a day, and on an average two quarts for a lactation period of from four to six months. This milk contains almost precisely the same proportion of fat as human milk, and its condemnation for nursery use by some authorities is declared to be a laboratory deliverance opposed to all practical experience. The suggestion that it should be more extensively employed, coming at a time when a cry of national degeneration from imperfect child-feeding is in the air, has the distinct merits of being practical in its character and easy of adoption by those who would be most affected by its results.

